# Supplementary material for: Differential Transcriptional Reprogramming by Wild Type and Lymphoma-Associated Mutant MYC Proteins as B-Cells Convert to a Lymphoma Phenotype
Source: Cancers (Basel). 2021 Dec 3;13(23):6093. doi: 10.3390/cancers13236093 (PMC8657136; doi:10.3390/cancers13236093)
Supplement: Supplementary file 1 [file cancers-13-06093-s001.zip › cancers-1459948-supplementary.pdf]

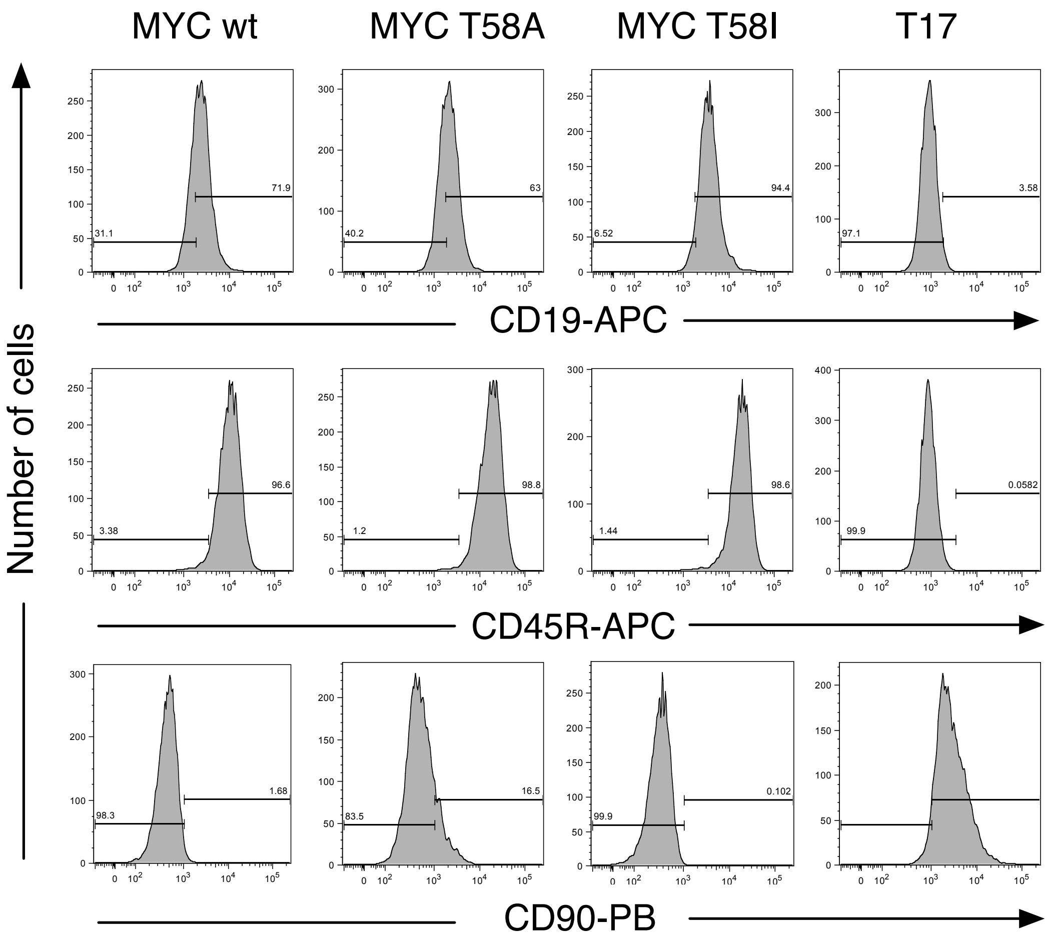

Supplementary Figure S1

LPS stimulated and subsequently transduced murine B-cells were positive for the B-cell markers CD19 and CD45R while negative for CD90, which is not expressed in B-cells, as measured by flow cytometry. The T-Cell line T17 was included as control.[1]

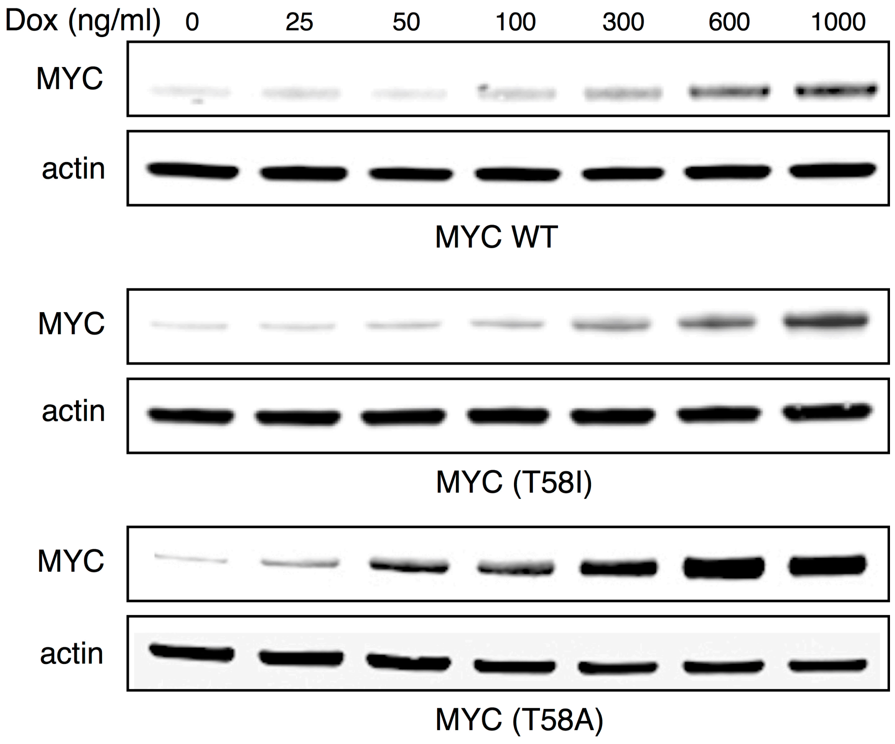

Supplementary Figure S2

MYC protein levels measured by western blot in murine B-cells expressing either WT MYC, T58A MYC or T58I MYC for increasing doxycycline concentrations in the culture media (0, 25, 50, 100, 300, 600, 1000 ng/ml).

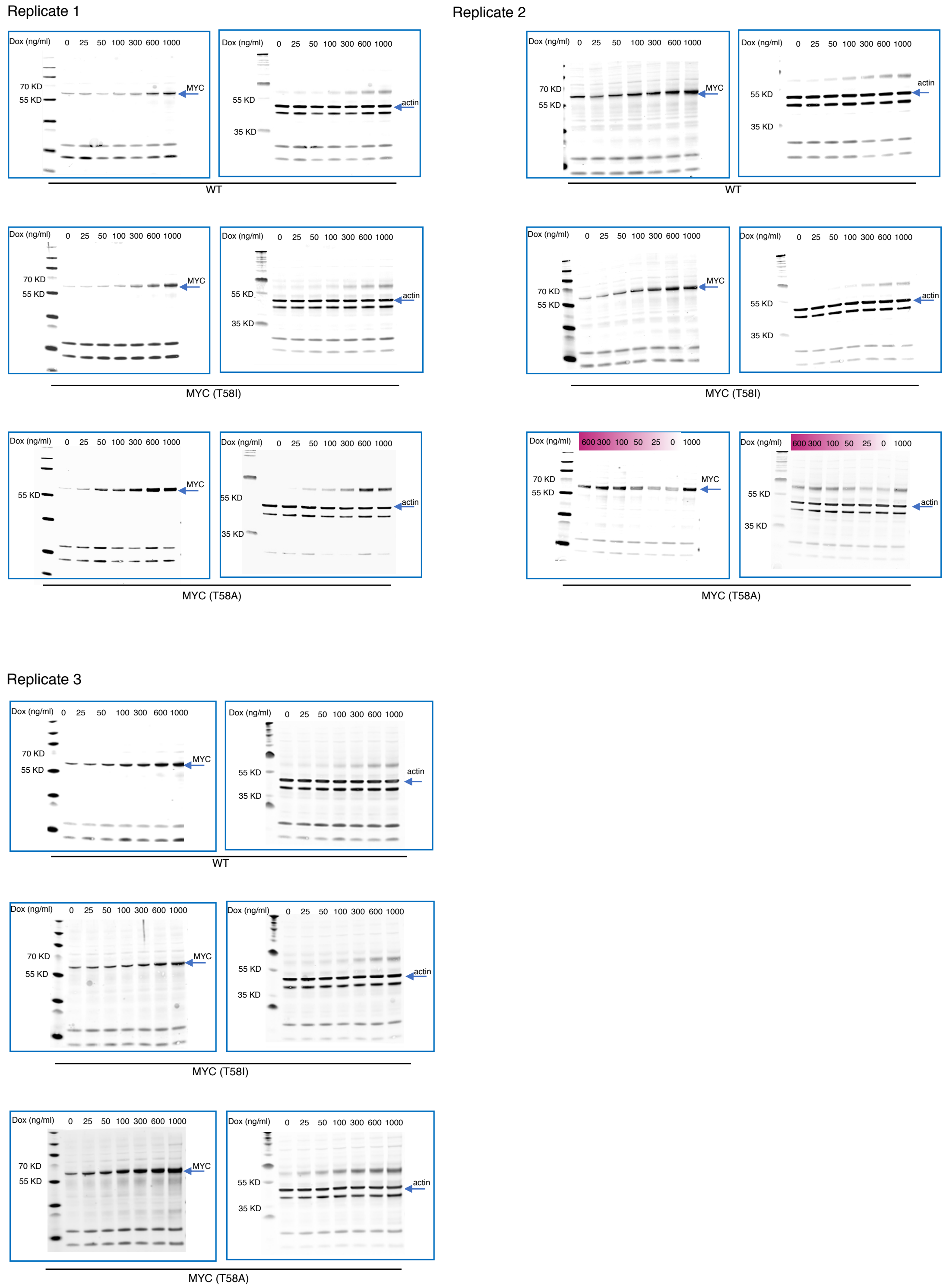

Supplementary Figure S3

Raw data for western blots shown in the paper. Figure 1B represents the quantification of 3 replicates shown here. Replicate 1 is raw data for figure presented in S2. In second replicate the loading of samples is altered as shown by color gradient.

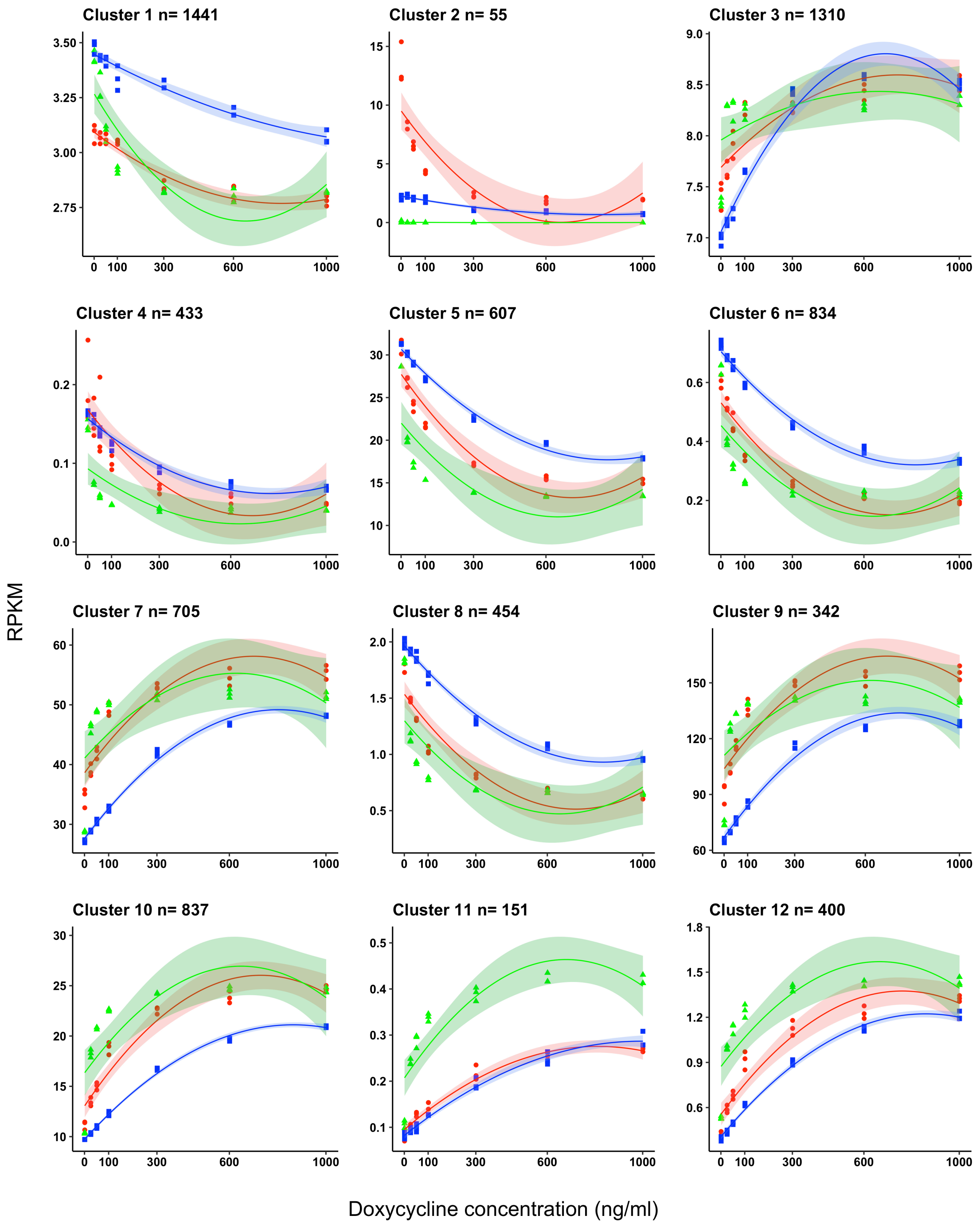

Supplementary Figure S4

Detailed view of median gene expression levels (RPKM) for 7569 clustered genes presented in Figure 3. WT MYC in red, T58A in green and T58I in red. The colored lines corresponding to the respective MYC genotype represent a 2nd degree polynomial regression model where the shaded areas indicate the 95% confidence interval for the model.

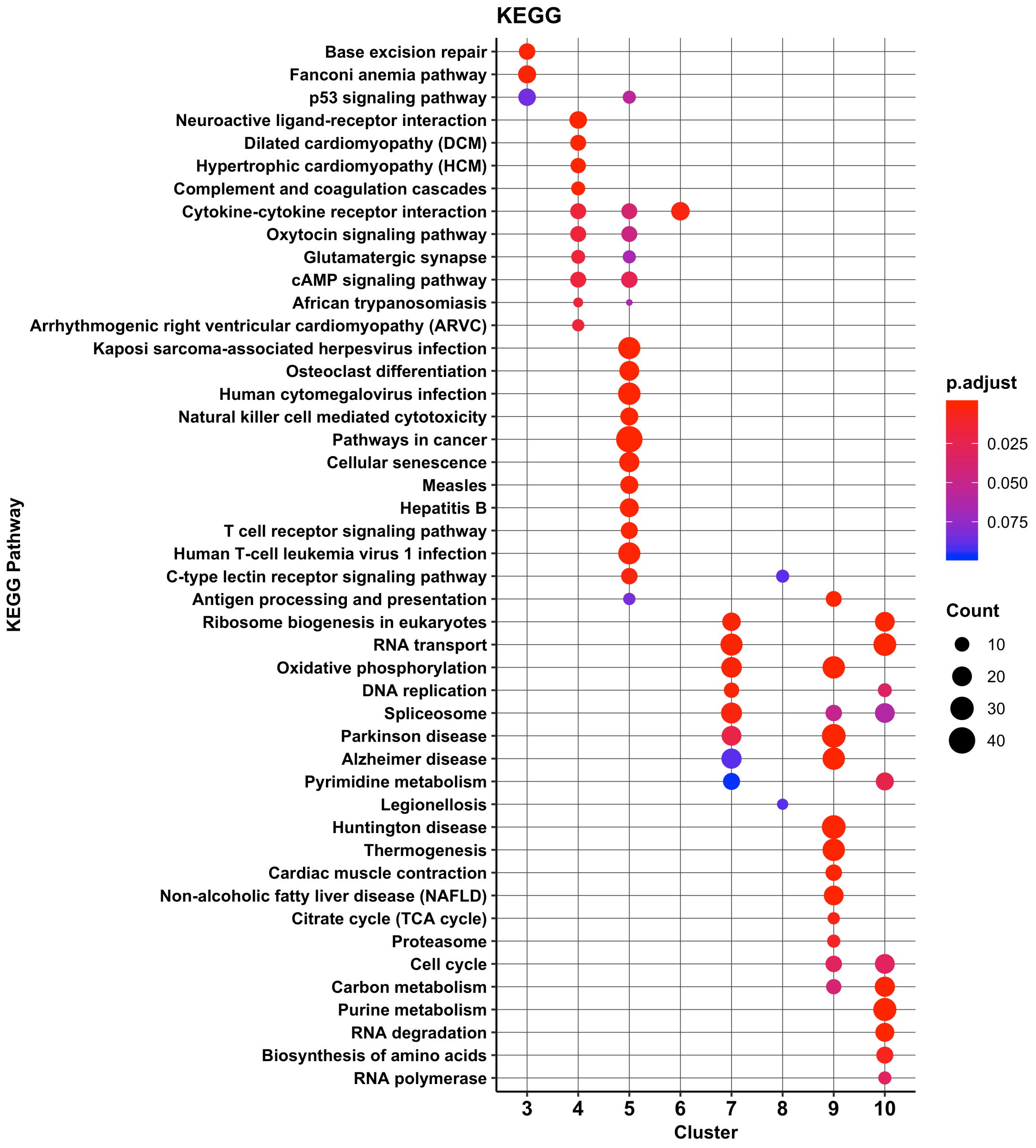

Supplementary Figure S5

Visualization of enrichment tests for clustered genes using the KEGG pathway database, the complete list of enriched gene sets is available in Supplementary Table S3.

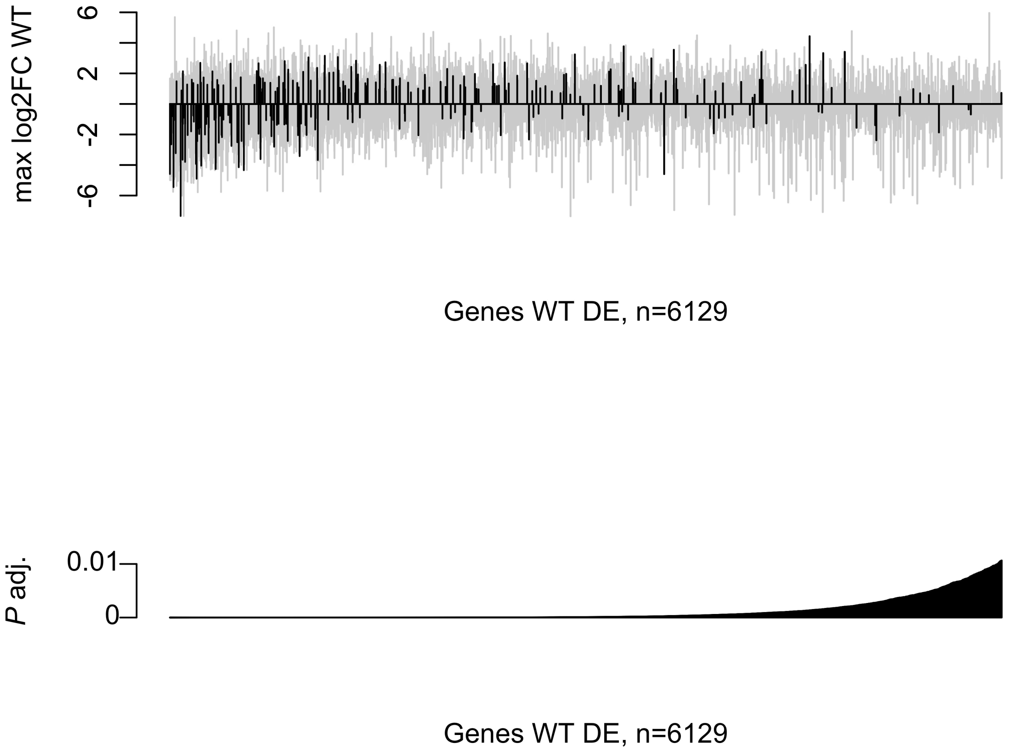

Supplementary Figure S6

Intersect between genes with significantly altered transcript levels upon MYC WT overexpression (gray bars) and direct MYC targets as identified by Zeller et al. (PNAS 2006) in the Burkitt's lymphoma cell line P493-6 (intersect: 294 genes, in black).[2] A significant enrichment of direct MYC target genes were observed for the most significantly differentially regulated genes upon MYC WT overexpression (Fisher's exact test *P*-value = 0.0011).

cluster	n genes in intersect	n expect	*P-value* fisher's exact test
1	0	14	4.00E-07
2	0	1	1
3	14	12	0.6
4	1	4	0.2
5	2	6	0.1
6	2	8	0.02
7	14	7	0.006
8	1	4	0.1
9	16	3	7.00E-08
10	18	8	8.00E-04
11	0	1	0.4
12	3	4	1

Supplementary Figure S7 Intersect between clusters with differentially expressed genes and direct MYC targets described by Muhar et al. (Science 2018).[3]

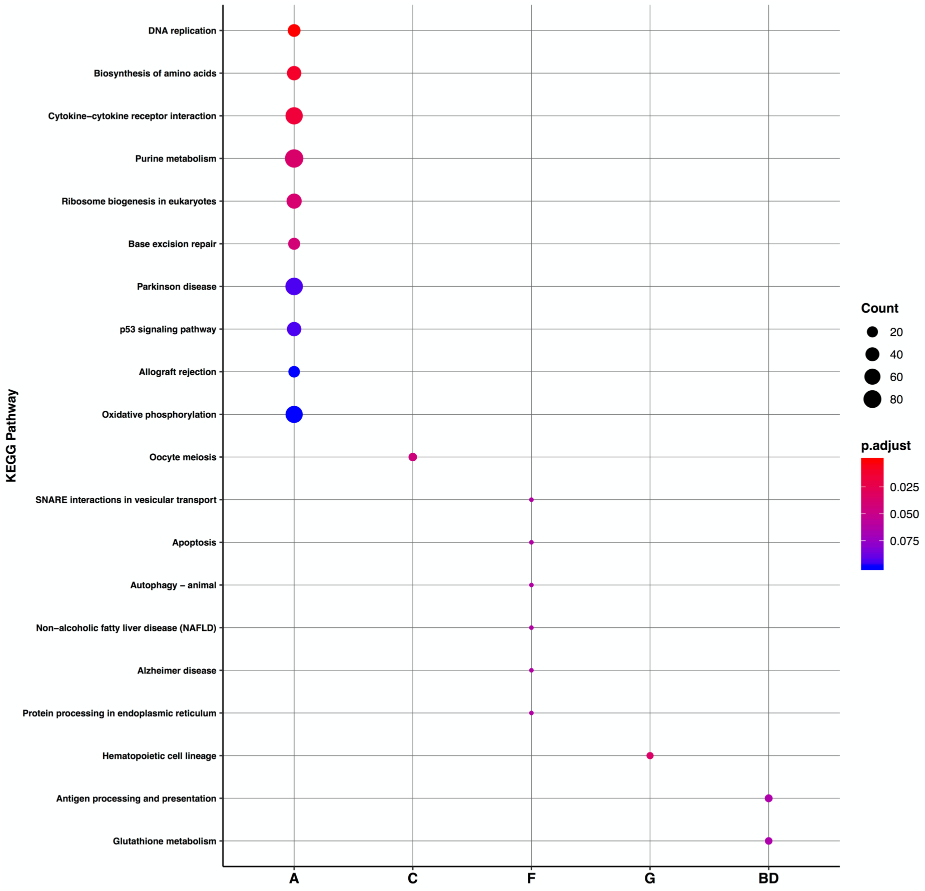

Supplementary Figure S8

Enrichment analysis for the Venn diagram subsets in figure 6A using the KEGG pathway database.

References to supplementary figures

1. Högstrand, K., Darmanin, S., Forshell, T. P. & Grandien, A. Transformation of mouse T cells requires MYC and AKT activity in conjunction with inhibition of intrinsic apoptosis. *Oncotarget* **9**, 21396–21410 (2018).

2. Zeller, K. I. *et al.* Global mapping of c-Myc binding sites and target gene networks in human B cells. *Proc Natl Acad Sci U S A* **103**, 17834–17839 (2006).

3. Muhar, M. *et al.* SLAM-seq defines direct gene-regulatory functions of the BRD4-MYC axis. *Science (80-.).* **360**, 800–805 (2018).
